# Effects of cooking methods on the iron and zinc contents in cowpea (*Vigna unguiculata*) to combat nutritional deficiencies in Brazil

**DOI:** 10.3402/fnr.v58.20694

**Published:** 2014-02-11

**Authors:** Elenilda J. Pereira, Lucia M. J. Carvalho, Gisela M. Dellamora-Ortiz, Flávio S. N. Cardoso, José L. V. Carvalho, Daniela S. Viana, Sidinea C. Freitas, Maurisrael M. Rocha

**Affiliations:** 1 School of Pharmacy, Universidade Federal do Rio de Janeiro, Rio de Janeiro, Brazil; 2 Embrapa Food Technology, Rio de Janeiro, Brazil; 3 Embrapa Mid-North, Av. Duque de Caxias, 5650. Teresina, Piauí, Brazil

**Keywords:** anemia, iron, zinc, cowpea, retention

## Abstract

**Background:**

Because iron deficiency anemia is prevalent in developing countries, determining the levels of iron and zinc in beans, the second most consumed staple food in Brazil, is essential, especially for the low-income people who experience a deficiency of these minerals in their diet.

**Objectives:**

This study aimed to evaluate the effect of cooking methods by measuring the iron and zinc contents in cowpea cultivars before and after soaking to determine the retention of these minerals.

**Methods:**

The samples were cooked in both regular pans and pressure cookers with and without previous soaking. Mineral analyses were carried out by Spectrometry of Inductively Coupled Plasma (ICP).

**Results:**

The results showed high contents of iron and zinc in raw samples as well as in cooked ones, with the use of regular pan resulting in greater percentage of iron retention and the use of pressure cooker ensuring higher retention of zinc.

**Conclusions:**

The best retention of iron was found in the BRS Aracê cultivar prepared in a regular pan with previous soaking. This cultivar may be indicated for cultivation and human consumption. The best retention of zinc was found for the BRS Tumucumaque cultivar prepared in a pressure cooker without previous soaking.

The populations of underdeveloped and developing countries generally suffer from malnutrition, which can cause illnesses from disability due to both calorie/protein and micronutrient deficiency (1). Iron and zinc are minerals that regulate important processes in the bodies of healthy individuals, and a deficiency of these minerals constitutes a public health concern, affecting mainly schoolchildren and women (2).

Iron deficiency in the diet can cause anemia, a disease still prevalent in the twenty-first century, affecting more than two billion people worldwide, mostly children and pregnant women (3). Iron deficiency is a condition in which there is a reduction in the total amount of iron and the iron supply is inadequate to meet the needs of different tissues, including the requirements for the formation of hemoglobin from erythrocytes (4). The recommended daily intake of dietary iron for normal infants is 1 mg per kg per day, for children it is 10 mg per day, and for adolescent men and women, it is 12 and 15 mg per day, respectively. Women during reproductive years require 15 mg per day, and adult men and postmenopausal women require only 10 mg per day (5).

According to the World Health Organization (WHO) in developing countries, 52% of pregnant women and 48% of children between 5 and 14 years of age are anemic, which means that approximately two million children in preschool are at risk of iron deficiency, which causes delays in mental development and, consequently, reduced attention span and learning (6).

On the other hand, zinc is an important mineral present in the human diet and its deficiency can affect normal growth and development of children, physical senses such as smell and taste, and can also result in anorexia and skin diseases (7). The percentage of the national population at risk for low zinc intake ranges from 68 to 95% in South and Southeast Asia, Africa, and the Eastern Mediterranean regions, and globally nearly half of the world's population is at risk for low zinc intake (8).

The second most consumed staple food in Brazil is beans, and it forms part of the daily menu of most of the population, providing important nutrients, such as protein, iron, zinc, and vitamins (9). The grains of beans are an important source of vegetable protein in the diet of the world population, especially in countries where the consumption of animal protein is limited for economic, religious, or cultural reasons. Beans are also the best vegetable source of iron (5.3–8.5 mg/100 g) and, therefore, an ally in combating nutritional deficiencies (10).

Cowpea (*Vigna unguiculata*) is an important component of diet in the developing countries of Africa, Latin America, and Asia and is also a valuable source of protein available at a low cost (11). In Northern and Northeastern regions of Brazil, cowpea is one of the foods consumed by low-income populations that suffer from malnutrition and micronutrient deficiencies, such as that of iron and zinc (3). Although there are government programs of iron and zinc supplementation, a large part of the population fails to benefit due to distribution problems in areas or regions with difficult access (12). Another strategy to combat these micronutrient deficiencies is the biofortification of cowpea. The improvement of new cultivars with high levels of iron and zinc can be an effective tool in combating iron deficiency anemia and in strengthening the immune system of the poor in Northeast Brazil (13).

Studies that have evaluated the influence of domestic cooking methods (regular pan cooking and pressure cooking) with and without previous soaking water on the retention of iron and zinc in beans are rare (14). Furthermore, there are no studies specifically in cowpea cultivars to assess the effect of cooking methods on retention of these minerals. Such studies are essential to indicate the most appropriate cooking method to retain iron and zinc to ensure that people, especially low-income individuals, have access to foods with high levels of these micronutrients that could improve their nutritional status. Because iron deficiency anemia is prevalent in developing countries, determining the levels of iron and zinc in beans is important, especially for the low-income population that suffers from deficiency of these micronutrients. In this study, the iron and zinc contents in raw and cooked cowpea cultivars were evaluated, before and after soaking, and the retention of these minerals was measured to recommend the best cultivar and cooking method for human consumption.

## Materials and methods

### Samples and materials

Five cowpea cultivars were used for this study: BRS Xiquexique, BRS Tumucumaque, BRS Aracê, BRS Guariba, and BR 17-Gurguéia (commercial). All the cultivars were grown in the same year in a field trial at Embrapa Mid-North, Teresina, Brazil (−5° 5″ S, 42° 48″ W), at an altitude of 72 m. The soil, an ultisol, was of sandy loam texture. Plowing, followed by disking, was carried out before sowing. For weed control, both preemergence (S-metolachlor) and postemergence (glyphosate) herbicides were used. Manual planting was undertaken, with four seeds placed per fosse/hole, followed by thinning 15 days after sowing, maintaining an average distance of 0.80 m between rows and 0.25 m between plants within a row. Two manual weedings using a hoe were performed during cultivation and pest control was carried out with dimethoate and thiamethoxan insecticides. Cultivation was realized in 2010, in the dry growing season (June–August) and in irrigation conditions of the sprinkler type (duration 2 h and irrigation interval of 5 days). The crop was harvested manually 75 days after sowing. The pods were dried in the sun, then machine threshed, and stored in plastic bags under refrigeration at 18°C.

### Cooking methods

Cowpeas of five bean cultivars were subjected to four different cooking methods: (1) regular pan cooking without previous water soaking; (2) regular pan cooking with previous water soaking; (3) pressure cooking without previous water soaking; and (4) pressure cooking with previous water soaking, using previously determined cooking times (Table 1). The beans were immersed in deionized water for 16 h, in capped pans, at room temperature. The grains were then cooked by exploiting water immersion.

**Table 1:** Cooking time for cowpea beans

		Cooking time (min)	
		
Cultivars	Cooking methods	NSW	WSW
BRS Xiquexique	RP	29′	8′
	PC	12′33″	4′3″
BRS Tumucumaque	RP	26′03″	5′03″
	PC	12′33″	4′3″
BRS Aracê	RP	32′	6′
	PC	11′6″	4′6″
BRS Guariba	RP	30′06″	11′03″
	PC	12′33″	2′6″
BR 17-Gurguéia	RP	31′	10′03″
	PC	13′3′	3′

RP=regular pan cooking; PC=pressure cooking; NSW=without soaking water; WSW=with soaking water.

To cook the grains not previously immersed in deionized water, distilled water was added to the beans (100 g): 500 mL of water. Cooking was carried out in commonly used, Teflon coated, semi-capped pot and pressure pan, both with a capacity of 3 L each.

The pots and lids were washed in water, immersed in a 5% nitric acid solution to decontaminate for more than 1 h, and rinsed with ultra-pure water (Milli-Q, Millipore, Milford, USA) before being subjected to the different cooking methods. Regular pan cooking was carried out with a half-open lid; 500 mL of boiling water was added during cooking to compensate for the loss of water that evaporated during cooking. No water replacement was necessary for the pressure-cooking method. Cowpea samples (100 g) were cooked in 500 mL of deionized water. All experiments were carried out in triplicate.

### Determination of Fe and Zn

All of the material was previously decontaminated in a nitric acid solution (HNO_3_ 1:1), prepared with distilled water. The raw bean samples were selected manually before being polished with a flannel cloth for 5 min and kept in clean glass receptacles. The polished bean grains were washed with deionized water (Milli-Q) and hand crushed. After washing, all of the water was discarded. Grain drying was performed in an oven overnight at 60°C, with no air circulation. The raw grains of each cultivar were grounded in a zirconium ball mill (RETSCH model MM200, Retsch Gmbtt & Co. KG. Haan, Germany), until a sufficient amount was obtained for analysis. The ground raw bean samples were divided into three aliquots of 0.2 g and placed in assay tubes. The broth in the cooked bean samples was completely drained through a plastic sieve into a beaker (previously weighed), and the bean grains were transferred into another glass receptacle, with three aliquots of 0.8 g of cooked grains and 2.0 g of broth being transferred into assay tubes. Sample digestion was carried out by acid hydrolysis, through the addition of 2 mL of nitric perchloric acid solution (2:1) for approximately 16 h at room temperature. After oxidation, the samples were heated in a digestion block (Technal, São Paulo, Brazil), in a fume hood at a slow boil to 100°C (±92°C) over 1 h and maintained for an additional 2 h at 170°C. After the samples were cooled at room temperature, 2 mL of the nitric perchloric acid solution was added to each tube, which was then returned to the digestion block for another 4 h at 170°C. The tubes were removed from the digestion block, and the samples were transferred into a volumetric flask (25 mL), and the volume was completed with ultra-pure water (Milli-Q).

Iron and zinc content in the samples of cooked and raw cowpea beans were determined by ICP Atomic Emission Spectrometry (Spectro Analytical Instruments – Spectroflame model P) (15).

### Moisture analysis

Determination of cooked cowpea moisture was carried out by the gravimetric method according to methodology developed by the Instituto Adolfo Lutz (16).

### Determination of the percentage retention of Fe and Zn

The iron and zinc retention percentage was determined based on the procedure described by Murphy, Criner, and Gray (17) and calculated by the following formula:

$$\text{Fe}/\text{Zn cooked grain}(\text{mg}.100{\text{g}}^{-1})=\frac{\left(\text{raw cowpea Fe or Zn})\times (\text{100}-\text{ cooked cowpea moisture}\right)}{\left(\text{100}-\text{ raw cowpea moisture}\right)}$$

Difference=(Fe/Zn mg.100 g^−1^in cooked beans, dry basis) – (Fe/Zn mg.100 g^−1^ in cooked beans, wet basis)

$$\text{Retention}(\%)=\frac{\text{Difference x 100}}{\text{Fe}/\text{Zn raw beans}(\text{ mg}.100{\text{g}}^{-1})}$$

## Statistical analysis

Results were expressed as means±standard deviations of three separate determinations. All data were treated by analysis of variance (ANOVA). Comparison among the treatment averages was by the least significance difference test (LSD) at the level of 5% of probability. All of the statistical analyses were carried out using Statistica software version 5.1.

## Results

Table 2 shows the results of the iron content measurements in five raw cowpea cultivars. The iron content varied from 6.4 (BRS Guariba) to 5.8 mg.100 g^−1^ (BR 17-Gurguéia). No significant differences (*P <* 0.05) were found either between BRS Xiquexique and BRS Tumucumaque or between BRS Aracê and BRS Guariba.

**Table 2:** Iron content (mg.100 g^−1^ dry matter) of five cowpea cultivars cooked in a regular pan and pressure cooked with and without soaking and iron retention

		Iron content (mg.100 g^−1^)				Iron retention (%)			
			
		Regular pan		Pressure cooking		Regular pan		Pressure cooking	
					
Cultivars	RB	NSW	WSW	NSW	WSW	NSW	WSW	NSW	WSW
BRS Xiquexique	5.1^a^	1.43^a^	1.74^a^	1.46^a^	1.25^b^	93.86±2.1^a^	98.22±1.2^b^	92.35±3.0^c^	91.57±1.3^c^
BRS Tumucumaque	5.1^a^	1.62^b^	1.55^b^	1.76^b^	1.60^b^	87.10±3.8^a^	94.58±1.1^b^	98.17±1.3^c^	97.05±1.6^c^
BRS Aracê	6.3^b^	2.42^c^	2.10^b^	2.26^c^	1.97^d^	94.08±0.2^a^	99.80±0.3^b^	92.54±0.6^c^	92.85±1.2^c^
BRS Guariba	6.4^b^	2.03^c^	2.13^c^	2.14^c^	1.93^c^	91.66±5.8^a^	96.53±2.2^a^	89.38±2.4^b^	96.53±0.9^c^
BR-17 Gurguéia	5.8^c^	1.99^d^	2.39^c^	1.91^d^	2.09^d^	97.18±4.2^a^	98.91±0.4^a^	92.47±4.4^b^	97.10±0.1^b^

RB=raw beans; NSW=without soaking water; WSW=with soaking water. Different letters within the same line differ significantly (5% significance level).

The iron content analyses of five cowpea cultivars cooked in a regular pan or pressure cooker with and without soaking are presented in Table 2.

There was significant difference in the iron content of BRS Xiquexique, BRS Tumucumaque, BRS Aracê, and BR-17 Gurguéia cultivars when cooked in a regular pan without prior soaking. The iron content of the beans that were cooked without soaking ranged from 2.42 (BRS Aracê) to 1.43 mg.100 g^−1^ (BRS Xiquexique).

On the other hand, when the previously soaked cowpeas were cooked in a pan, the iron content ranged from 2.39 (BR 17-Gurguéia) to 1.55 mg.100 g^−1^ (BRS Tumucumaque).

The iron contents of the cultivars that were cooked in a pressure cooker without soaking ranged from 2.26 (BRS Aracê) to 1.46 mg.100 g^−1^ (BRS Xiquexique). After being previously soaked, the iron content of the cowpeas ranged from 2.09 (BR 17-Gurguéia) to 1.25 mg.100 g^−1^ (BRS Xiquexique).

Immersion was not effective at increasing the iron content when cooked in the regular pan, since the increase in the level of iron was negligible in the BR-17 Gurguéia cultivar.

In a pot under pressure, the only significant difference was a reduction in the iron content of the BRS Aracê cultivar observed among the treatments with and without previous soaking. No other significant differences were found between the cooking methods (in a regular pan and in a pot under pressure) or between beans that had been soaked and those that were not (*P*>0.05).

Results show that the most effective procedure was cooking in a regular pan without previous soaking and that BRS Aracê cultivar presented the best iron contents.

Table 3 shows the zinc contents of the cowpea cultivars cooked with and without previous soaking in a regular pan and in a pressure cooker.

**Table 3:** Zinc content (mg.100 g^−1^ dry matter) of five cowpea cultivars cooked in a regular pan and pressure cooked without and with soaking and zinc retention

		Zinc content (mg.100 g^−1^)				Zinc retention (%)			
			
		Regular pan		Pressure cooking		Regular pan		Pressure cooking	
					
Cultivars	RB	NSW	WSW	NSW	WSW	NSW	WSW	NSW	WSW
BRS Xiquexique	3.5^d^	1.29^a^	1.44^a^	1.18^a^	1.12^a^	86.17±2.6^a^	90.20±2.1^a^	92.02±2.6^b^	97.22±2.9^b^
BRS Tumucumaque	3.8^b^	1.25^a^	1.23^a^	1.49^b^	1.32^b^	87.21±3.6^a^	96.41±0.9^b^	99.73±0.3^c^	99.05±1.2^c^
BRS Aracê	4.5^c^	1.93^b^	1.63^c^	1.89^d^	1.55^c^	96.54±0.7^a^	98.64±0.4^b^	99.18±1.1^c^	96.34±1.6^c^
BRS Guariba	3.6^d^	1.28^a^	1.34^a^	1.44^c^	1.25^d^	93.44±5.1^a^	98.82±1.9^a^	96.13±3.8^d^	97.77±0.2^d^
BR-17 Gurguéia	4.0^e^	1.35^c^	1.75^d^	1.51^c^	1.43^e^	94.65±4.1^a^	98.32±0.2^b^	93.46±2.9^c^	95.81±1.5^c^

RB=raw beans; NSW=without soaking water; WSW=with soaking water. Different letters within the same line differ significantly (5% significance level).

The zinc content of the beans that were cooked in a regular pan without prior soaking ranged from 1.93 (BRS Aracê) to 1.25 mg.100 g^−1^ (BRS Tumucumaque). The zinc content of the beans that were soaked before cooking varied from 1.75 (BR 17-Gurguéia) to 1.23 mg.100 g^−1^ (BRS Tumucumaque)

When pressure cooked without previous immersion, the zinc content ranged from 1.89 (BRS Aracê) to 1.18 mg.100 g^−1^ (BRS Xiquexique). In a pressure cooker with previous immersion, the zinc content ranged from 1.55 (BRS Aracê) to 1.12 mg.100 g^−1^ (BRS Xiquexique). The results revealed no significant differences (*P*<0.05) between the BRS Xiquexique and BRS Tumucumaque cultivars or between BRS Guariba prepared in the common pan with and without immersion. In the pressure cooker, the results differed significantly (*P*<0.05) between the soaked and non-soaked beans for the BRS Aracê, BRS Guariba, and BR 17-Gurguéia cultivars (*P*<0.05) but not the BRS Xiquexique and BRS Tumucumaque cultivars.

Table 3 shows the effect of the cooking method on zinc retention in cowpea beans.

The percentage of zinc retention was high for all treatments, indicating that the cooking methods were effective in the preservation of this micronutrient. The retention of zinc was higher after the immersion treatment for all of the cultivars when prepared in the regular pan (*P <* 0.05); however, there was no statistically significant difference when the samples were prepared in the pressure cooker. The retention of zinc was higher when the cowpeas were prepared in the pressure cooker compared to the regular pan for the BRS Xiquexique, BRS Tumucumaque, and BRS Aracê cultivars (*P <* 0.05), while there was no significant difference for the BRS Guariba and BR 17-Gurguéia cultivars. The best retention of zinc was found for the BRS Tumucumaque cultivar (99.73±0.3%) prepared in the pressure cooker without previous soaking.

## Discussion

According to Moreira-Araújo et al. (18), a content of 4.5 mg.100 g^−1^ of iron was found in the cowpea cultivar BR3-Tracuateua, a value lower than the one found in our studies of 6.4 mg.100 g^−1^ for BRS Guariba. A study by Brito et al. (6) indicated that beans are the main source of iron in the diet of individuals aged 7–17 years who do not consume red meat. Although animal products enhance iron absorption, they are considered costly, which may limit their consumption in low-income populations.

The data also show that the zinc concentrations in the five cultivars varied from 4.4 (BRS Aracê) to 3.8 mg.100 g^−1^ (BRS Tumucumaque) (Table 3). The zinc concentrations differed significantly among all the examined cowpea cultivars (*P*<0.05). Carvalho et al. (19) observed that in cowpea, the lowest level of zinc was found to be 2.7 mg.100 g^−1^, which was less than what was found in our studies.

Mean daily dietary zinc intake of populations from several countries range from 4.7 to 18.6 mg.100 g^−1^. International studies have found that zinc deficiency can also be a common health concern in developing countries where the consumption of animal protein is low (20).

Soaking has been shown to cause the loss of important vitamins and minerals that are eliminated with the discarded soaking solutions (21). Lestienne et al. (22) analyzed the content of iron in cowpea with and without previous soaking and noted a reduction in the iron content after the immersion treatment. However, these results were evaluated in the raw grains, whereas in our studies, the influence of soaking on the iron content was observed after heat treatment. Because the beans are typically consumed as a cooked product, the effect of soaking on the iron content after heat treatment is important.

Lestienne et al. (22) assessed the zinc content in cowpea cultivars cooked in a pan with and without soaking. They observed that the zinc content of the beans increased after soaking in water. Previous studies indicate soaking may reduce the mineral content that may be partially or completely removed with solubilization and after discarding the soaking water (23); however, these minerals may be retained in the broth.

According to Miller (24), loss of minerals during cooking is not caused by destruction but by leaching (extraction of minerals by water) into the cooking water. To minimize energy costs, the soaking of cowpea can be recommended as no nutritional loss was observed in other studies (25).

According to Taiwo (26), long cooking times use more fuel and can result in a loss of nutrients, which constrains the use of dry beans as food in developing countries. Cooking time of beans can be quite long, because it depends on the variety as well as on the cooking process. Soaking prior to cooking has been found to influence both the texture of the cooked beans and the cooking time. Cowpea grain can be cooked quickly, which is an important consideration in developing countries which experience shortage of fuel.

The percentage of iron retention was high for all treatments, indicating that the cooking methods were effective in the preservation of this micronutrient (Table 2).

Comparing the results of cooking in a regular pan with and without soaking, the retention of iron was higher after the immersion treatment in the BRS Xiquexique, BRS Tumucumaque, and BRS Aracê cultivars, while in the pressure cooker, there was no statistically significant difference among cultivars (*P <* 0.05).

The retention of iron was higher in the regular pan compared to the pressure cooker for the BRS Xiquexique, BRS Aracê, and BR 17-Gurguéia cultivars. The best retention of iron was found in the BRS Aracê cultivar (99.80±0.3%) prepared in a regular pan with previous soaking. This cultivar contained 6.34 mg.100 g^−1^ of iron in the raw grain and also required a shorter cooking time without soaking in the pressure cooker; this cultivar may be recommended for cultivation and human consumption.

Despite being a common practice in Brazil to leave beans soaking and to cook beans in a pressure cooker to reduce the cooking time, the results of this study showed that the retention of iron in cowpea was higher when prepared in a regular pan. In our study, shorter cooking times showed a trend toward a lower percentage of retention of iron. This finding has also been observed for the retention of B group vitamins in meat (27). According to *Codex Alimentarius*, a food can be labeled as ‘source’ of a nutrient when 100 g of the product presents more than 15% of the dietary reference intake (DRI) of the desired nutrient. Similarly, when the food contains at least the double of the amount determined as ‘source’, it can be labeled ‘high’ (28). For example, to be considered as source a food must provide a 15% DRI which for iron is equivalent to 21 µg/g product (2.1 mg.100 g^−1^) and for zinc is 22.5 µg/g (2.25 mg.100 g^−1^), and is regarded as having a ‘high’ content when it contains 42 µg of iron/g product and 45 µg of zinc/g of product. The presence of phytates and tannins in vegetable fibers can negatively influence the bioavailability of zinc and iron as cereals and pulses. However, the tannins have no significant influence on the bioavailability of zinc and iron from cereals and legumes (29). Phytic acid is reduced after processes such as soaking, germination, and heat, promoting an increase in mineral extractability and bioavailability.

Thus, the cowpea bean was confirmed to be an excellent source of minerals for human consumption. Taking into account the fact that the majority of the world population experience iron and zinc deficiency, the high contents of both these minerals found mainly in BRS Guariba and BRS Aracê cultivars confirm the fact that the cowpea bean can be regarded as a good source of both iron and zinc. Thus, these cultivars can be used to help fight anemia in low-income populations of the underdeveloped and the developing countries, especially in infants and pregnant women.

The importance of beans is so great that the Ministry of Health encourages the consumption of legumes and includes the intake of beans as part of one of the 10 steps to a healthy diet listed in the Brazilian Food Guide. The main international bodies that encourage and promote health also recommend a daily intake of one or more servings of beans (30).

## Conclusions

Cowpea is an excellent source of iron and zinc in raw grains and can be used for the low-income population in Brazil who suffer from a deficiency of these micronutrients. The immersion of grains prior to cooking is already a common practice among Brazilian housewives; however, the results of the present study demonstrate that this method had no effect on the concentrations of iron and zinc. Nevertheless, the best retention of iron was found in the BRS Aracê cultivar prepared in a regular pan with previous soaking, showing that this cultivar may be recommended for cultivation and human consumption.

The retention of zinc was higher when the cowpeas were prepared in the pressure cooker compared to the regular pan for the BRS Xiquexique, BRS Tumucumaque, and BRS Aracê cultivars. The best retention of zinc was found for the BRS Tumucumaque cultivar prepared in the pressure cooker without previous soaking.

Traditional methods of cooking of cowpea in Brazil (in a regular pan or pressure cooker and with or without previous soaking) proved to be efficient for the maximum retention of iron and zinc.

Because the cost of a pressure cooker is prohibitive for low-income populations, cooking the cowpea in a common pot minimizes the cost and allows these groups to have access to foods that have a high percentage of these micronutrients.
